# Case Report: Inflammatory CADASIL phenotype associated with a rare cysteine-sparing NOTCH3 variant

**DOI:** 10.3389/fnhum.2025.1568937

**Published:** 2025-05-14

**Authors:** Abraham Madjidov, Mary-Alice Abbott, Katherine M. Mullen, Richard Hicks, Gottfried Schlaug

**Affiliations:** ^1^Department of Neurology, Baystate Medical Center, University of Massachusetts Chan Medical School - Baystate, Springfield, MA, United States; ^2^Department of Pediatrics, Baystate Medical Center, University of Massachusetts Chan Medical School - Baystate, Springfield, MA, United States; ^3^Department of Radiology, Baystate Medical Center, University of Massachusetts Chan Medical School - Baystate, Springfield, MA, United States

**Keywords:** CADASIL, inflammation, case report, NOTCH3, variant of uncertain significance, cysteine-sparing

## Abstract

We present a 50-year-old female who has a longstanding history of migraine with aura. She experienced one episode of partial visual field loss associated with a small acute/subacute lesion involving the cortex and subcortical white matter (showing contrast enhancement), alongside extensive white matter hyperintensities. Given these findings, CADASIL was suspected. Genetic testing identified a rare heterozygous *NOTCH3* variant (c.6102dup, p.Gly2035Argfs*60), currently classified as a variant of uncertain significance. Concurrent cerebrospinal fluid analysis revealed elevated myelin basic protein, an elevated IgG index, and 4 oligoclonal bands, indicating an inflammatory process. Her visual evoked potentials showed no evidence of optic nerve or tract impairment. Approximately 9 months later, the occipital lesion evolved into encephalomalacia and gliosis without enhancement, while the extensive white matter hyperintensities remained stable. A repeat lumbar puncture 1 year later showed persistently elevated myelin basic protein and IgG index, now with 7 oligoclonal bands (some shared with serum). Currently, her neurological examination is normal. She is managed on dual antiplatelet therapy, and her migraines are effectively controlled with calcium-channel blocker prophylaxis. Notably, her mother, diagnosed with multiple sclerosis for several decades despite imaging findings suggestive of CADASIL, shares the same *NOTCH3* variant. One of her two children tested negative for the variant and had normal imaging, while the other minor child has a significant history of migraines with aura. Our patient’s clinical presentation and comprehensive findings raise the possibility of an inflammatory phenotype potentially associated with the rare *NOTCH3* c.6102dup variant, though causation remains unclear. Coexistence with another demyelinating central nervous system disease is possible, and further research is needed to clarify this relationship. If inflammatory variants of CADASIL exist, alternative treatments targeting inflammation may need consideration.

## Introduction

Cerebral Autosomal Dominant Arteriopathy with Subcortical Infarcts and Leukoencephalopathy (CADASIL) is the leading hereditary cause of non-arteriosclerotic, amyloid-negative small vessel disease (Joutel et al., 1996). Symptoms typically begin in the 3rd or 4th decade and include migraines with aura, recurrent small strokes (often clinically silent), mood disorders, cognitive impairment, and eventual dementia (Chabriat et al., 2009). The prevalence is estimated at 2–5 per 100,000, affecting males and females equally, with some population variation (Chabriat et al., 2009; Moreton et al., 2014; Narayan et al., 2012; Razvi et al., 2005).

CADASIL is an autosomal dominant disorder caused by pathogenic variants in *NOTCH3*, which encodes the NOTCH3 transmembrane protein (Joutel et al., 1996). Nearly all known pathogenic variants in CADASIL disrupt the number of cysteine residues within the epidermal growth factor-like repeat (EGFr) domains of NOTCH3’s extracellular domain. This disruption affects the disulfide bond structure, causing misfolding of the NOTCH3 protein (Chabriat et al., 2009). Misfolded NOTCH3 extracellular domain accumulates in small artery walls, forming granular osmiophilic material (GOM) deposits, a hallmark of CADASIL detectable via skin biopsies (Ruchoux et al., 1995). This accumulation results in vascular smooth muscle cell (VSMC) degeneration, arterial wall thickening, luminal stenosis, and impaired blood flow (Tikka et al., 2014).

A CADASIL diagnosis is confirmed through genetic testing or by finding GOM deposits on skin biopsy (Mancuso et al., 2020). Differentiating CADASIL from inflammatory or autoimmune disorders, such as multiple sclerosis (MS), can be challenging. Some patients with CADASIL show inflammatory markers, raising the possibility of an “inflammatory CADASIL” phenotype or coexistence with concurrent autoimmune conditions (Schiess et al., 2018).

## Case description

**FIGURE 1:**
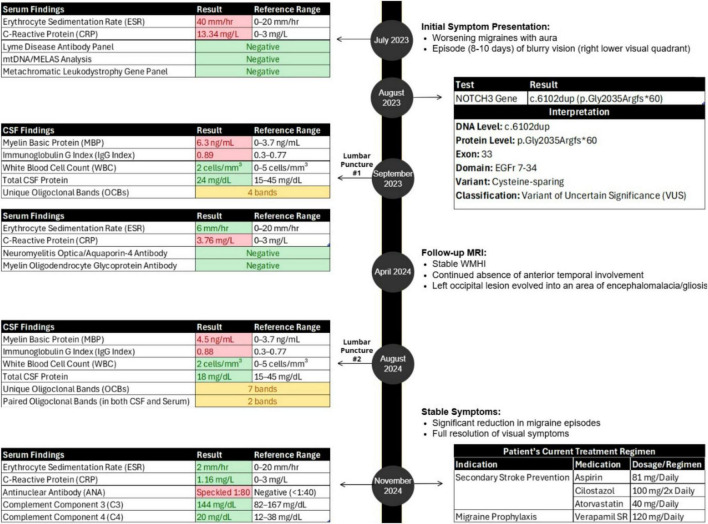
Case report timeline.

**FIGURE 2:**
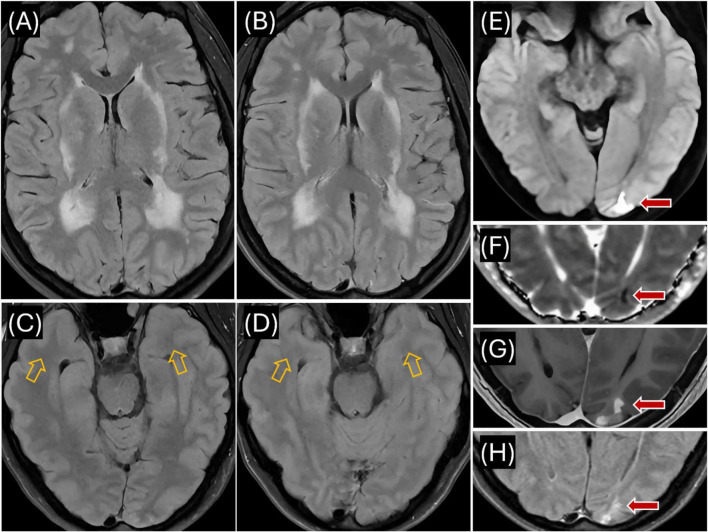
Proband’s MR imaging. **(A)** Axial T2 FLAIR (July 2023) showing extensive hyperintensities in the periventricular and deep white matter, including bilateral external capsules, indicative of CADASIL-related changes; **(B)** Axial T2 FLAIR (April 2024) demonstrating persistent hyperintensities in periventricular and deep white matter, similar to July 2023 findings; **(C)** Axial T2 FLAIR (July 2023) showing no signal abnormality in the anterior temporal lobe white matter (see orange arrows); **(D)** Axial T2 FLAIR (April 2024) confirming continued absence of hyperintensities in the anterior temporal lobes (see orange arrows); **(E)** Axial DWI (July 2023) revealing reduced diffusivity in the left occipital lobe (see red arrow); **(F)** Axial ADC map (July 2023) demonstrating isointense to minimally hypointense signal in the left occipital lesion, consistent with acute/subacute ischemia (see red arrow); **(G)** Axial T1 with contrast (July 2023) showing patchy contrast enhancement in cortex and juxtacortical white matter of the left occipital region (see red arrow); **(H)** Axial T2 FLAIR (April 2024) showing evolution of the left occipital lesion into a 1.4 cm area of encephalomalacia and gliosis (see red arrow).

In July 2023, a 50-year-old female presented with worsening migraines (with aura) over the past year and a recent 8–10 day episode of blurry vision in the right lower visual field quadrant (Figure 1). Initial imaging (CT, CTA, CTV) was normal. Subsequent MRI revealed extensive white matter hyperintensities (WMHI) in bilateral subcortical and corona radiata white matter regions (Figure 2A). Characteristically for CADASIL, WMHI were also present in the external and extreme capsules, along with lacunar infarcts in the thalamus and basal ganglia (Figure 2A), without anterior temporal lobe involvement (Figure 2C). An acute/subacute lesion in the left posterior occipital lobe (Figure 2E) demonstrated isointense signals with areas of reduced diffusivity involving the cortex and juxtacortical white matter, accompanied by associated T2 prolongation (Figure 2F). Patchy and curvilinear enhancement was observed along the cortex and juxtacortical white matter (Figure 2G).

Genetic testing in August 2023 identified a rare heterozygous *NOTCH3* variant (c.6102dup, p.Gly2035Argfs*60), currently classified as a variant of uncertain significance (VUS) (Figure 1) (Landrum et al., 2013).

Our patient’s inflammatory serum markers were initially elevated, with an erythrocyte sedimentation rate (ESR) of 40 mm/hr (normal range: 0–20 mm/hr) and C-reactive protein (CRP) level of 13.34 mg/L (normal range: 0–3 mg/L); these normalized after 6 weeks (ESR 6 mm/hr, CRP 3.76 mg/L) (Figure 1). Cerebrospinal fluid (CSF) analysis via lumbar puncture revealed elevated myelin basic protein (MBP, 6.3 ng/mL; normal range: 0–3.7 ng/mL), an elevated IgG index (0.89; normal range: 0.3–0.77), and the presence of 4 oligoclonal bands (OCBs) unique to CSF (Figure 1). CSF white blood cell count was normal (2 cells/mm^3^; normal range: 0–5 cells/mm^3^), and total CSF protein count was within normal limits (24 mg/dL; normal range: 15–45 mg/dL). Serum testing for Lyme antibodies, mitochondrial variants (mtDNA analysis/MELAS), and a metachromatic leukodystrophy gene panel (ARSA, PSAP, SUMF1) was negative (Figure 1).

Follow-up MRI in April 2024 showed stable WMHI (Figure 2B), again without anterior temporal lobe involvement (Figure 2D). The previous occipital lesion evolved into a 1.4 cm area of encephalomalacia and gliosis (Figure 2H).

A repeat lumbar puncture in August 2024 confirmed ongoing inflammation with elevated MBP (4.5 ng/mL), an elevated IgG index (0.88), and 7 unique CSF OCBs (Figure 1). Additionally, 2 paired OCBs were identified in both CSF and serum. CSF analysis also consistently showed a normal white blood cell count (2 cells/mm^3^) and normal protein count (18 mg/dL), indicating the absence of active infection or demyelination. NMO/Aquaporin4 and MOG antibodies were also negative (Figure 1).

Blood work in November 2024 revealed normal ESR (2 mm/h) and CRP (1.16 mg/L), positive ANA (speckled pattern, 1:80), and normal complement levels, including C3 (144 mg/dL; normal range: 82–167 mg/dL) and C4 (20 mg/dL; normal range: 12–38 mg/dL) (Figure 1).

**FIGURE 3:**
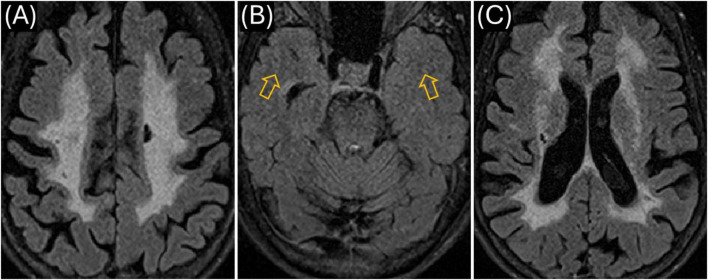
Mother’s MR imaging (obtained March 2023). **(A)** Axial T2 FLAIR showing extensive hyperintensities in the centrum semiovale region bilaterally; **(B)** similar to the Proband, there was no signal abnormality in the anterior temporal lobe white matter (see orange arrows); **(C)** WMHI in the external and extreme capsule on both sides as well as periventricular regions on both sides.

We also identified the same *NOTCH3* VUS in the patient’s mother (currently aged 73, diagnosed with MS in her 50s). However, most recent and previous MRIs showed a WMHI distribution mostly in the corona radiata/subcortical white matter (Figures 3A, C), more consistent with CADASIL, although the same sparing of the white matter in the anterior temporal lobes (Figure 3B) as in the index patient. CSF studies performed over 20 years ago are no longer available, but her neurologist (via oral communication) confirmed those results did not strongly support an MS diagnosis. Nevertheless, the mother received immune-modulating medications for presumed MS over many years, yet experienced progressive cognitive and motor decline. The index patient’s eldest male child, aged 20, tested negative for the *NOTCH3* mutation and had no MRI abnormalities. A younger, non-adult female child has not been tested but has a strong history of migraine headaches.

Since her initial presentation, our patient’s visual symptoms have resolved. No evidence of optic nerve demyelination on visual evoked potentials was seen, and no hypercoagulable conditions have been identified. She is currently managed with aspirin, cilostazol, and atorvastatin for secondary stroke prevention, and verapamil as a migraine prophylaxis medication, significantly reducing both the frequency and severity of her migraines (Figure 1).

## Discussion

The *NOTCH3* c.6102dup (p.Gly2035Argfs*60) variant currently remains classified as a VUS (Landrum et al., 2013). However, our findings provide preliminary evidence supporting its potential pathogenicity. Our patient’s clinical presentation, including migraines with aura, visual field deficits, and extensive WMHI in a characteristic distribution, aligns with established clinical criteria for CADASIL (Chabriat et al., 2009). Further support for pathogenicity comes from a Finnish CADASIL cohort study that identified the same *NOTCH3* c.6102dup variant in one patient and provided two key pieces of evidence linking this variant to CADASIL (Mönkäre et al., 2022). First, a skin biopsy from that patient showed GOM deposits. Finding GOM deposits associated with this variant supports its pathogenic potential (Ruchoux et al., 1995). Second, segregation analysis in the same family showed that the patient’s healthy mother and sibling did not carry the c.6102dup variant, consistent with the autosomal dominant inheritance pattern characteristic of CADASIL (Joutel et al., 1996).

We conducted bioinformatic analyses to further assess the potential pathogenicity of the NOTCH3 c.6102dup variant. This variant is exceedingly rare, with an allele frequency of approximately 0.000006877 (7 per million individuals) (Karczewski et al., 2020). Such low frequency aligns with expectations for pathogenic variants, as natural selection typically limits their prevalence (Karczewski et al., 2020; Richards et al., 2015). The variant’s Combined Annotation Dependent Depletion (CADD) score of 28.0 places it among the top 0.1%–1% most deleterious genetic variants across the human genome, indicating substantial pathogenic potential (Kircher et al., 2014). Moreover, the affected genomic region has a high evolutionary conservation score of 8.68 (PhyloP; range −20 to 9.631), suggesting it serves an important functional role in the NOTCH3 protein. Therefore, disruptions in this genomic region may significantly increase pathogenic risk (Pollard et al., 2009).

Complementing these bioinformatic findings, *in silico* analyses (SpliceAI: 0.00; Pangolin: 0.0700) show a minimal probability of altered RNA splicing for the c.6102dup variant (Jaganathan et al., 2019; Zeng and Li, 2022). These results support that nucleotide duplication at DNA position 6102 is the probable pathogenic mechanism. This duplication causes a frameshift, introducing a premature stop codon predicted to result in a truncated NOTCH3 protein that may impair VSMC function (Mönkäre et al., 2022). Furthermore, the variant is cysteine-sparing and occurs in exon 33 within EGFr domains 7–34. Variants within these domains generally cause milder clinical symptoms, later onset of stroke, slower disease progression, and greater clinical variability compared to variants located in EGFr domains 1–6 (Rutten et al., 2018). Consistent with these observations, imaging studies of patients carrying cysteine-sparing *NOTCH3* variants show decreased anterior temporal lobe white matter involvement, with 91% of systematically reviewed cases showing no such involvement (Muiño et al., 2017). Our patient’s MRIs consistently showed an absence of anterior temporal lobe white matter involvement (Figures 2C, D), aligning with the imaging characteristics typical of cysteine-sparing *NOTCH3* variants (Muiño et al., 2017). Additionally, her relatively mild symptoms, clinical improvement with treatment, and observed clinical variability (potentially reflecting ongoing inflammatory processes) align with characteristics of variants located in EGFr domains 7–34 (Rutten et al., 2018). While current evidence supports the potential pathogenicity of this variant, further validation through functional assays, additional segregation analyses within affected families, or identification of GOM deposits remains necessary for definitive classification according to established guidelines (Richards et al., 2015).

The c.6102dup *NOTCH3* variant identified in our patient might be associated with an inflammatory phenotype; however, additional cases or functional studies are needed to establish a definitive link. Our patient presented with several notable inflammatory features in the CSF, including a persistently elevated IgG index (initially 0.89, and 0.88 at 1-year follow-up), a progressive increase in OCBs from 4 to 7 bands over the same period, and elevated MBP levels (initially 6.3 ng/mL, slightly declining to 4.5 ng/mL at 1-year follow-up). Additionally, we detected 2 paired OCBs shared in both CSF and serum (Figure 1). This pattern is atypical for MS, where OCBs are usually restricted to CSF and absent from serum (McGinley et al., 2021). We also closely monitored serum inflammatory markers, which were initially elevated, showing an ESR of 40 mm/h (normal: 0–20 mm/h) and CRP of 13.34 mg/L (normal: 0–3 mg/L). However, both ESR and CRP rapidly declined, reaching near-normal levels within 6 weeks (ESR: 6 mm/h, CRP: 3.76 mg/L), and fully normalizing by November 2024 (ESR: 2 mm/h, CRP: 1.16 mg/L). Furthermore, the patient intermittently showed mild signs of systemic autoimmunity, including a low-titer antinuclear antibody (ANA) at 1:80 with a speckled pattern (Figure 1). Complement levels remained normal (C3: 144 mg/dL [normal: 82–167 mg/dL], C4: 20 mg/dL [normal: 12–38 mg/dL]). The low-titer ANA finding was non-specific and not associated with any defined autoimmune disease.

We performed a thorough diagnostic workup to exclude alternative inflammatory or autoimmune conditions, including Lyme disease (negative serology), MS, neuromyelitis optica (NMO), myelin oligodendrocyte glycoprotein (MOG) antibody-associated disorders (negative antibody tests), mitochondrial disorders (absence of pathogenic mitochondrial DNA mutations), and Fabry disease (normal alpha-galactosidase activity). Additionally, tests for common viral infections (influenza, RSV, COVID-19) were negative, and the patient denied any recent infections. Neuroimaging provided further clarity, as her brain MRI lacked characteristic features commonly associated with MS or other neuroinflammatory conditions, such as Dawson’s fingers, optic neuritis (normal VEP), perivenular enhancement, or vessel-wall enhancement (McGinley et al., 2021). Despite negative findings, subtle or atypical autoimmune processes or environmental factors might still contribute to our patient’s clinical presentation. Paraskevas et al. (2018) described several autoimmune disorders co-occurring with CADASIL, including MS, central nervous system (CNS) angiitis, autoimmune thrombocytopenia, and renal involvement with IgA mesangial deposits (Paraskevas et al., 2018). Additionally, environmental triggers may also unmask or exacerbate CADASIL; Król et al. (2022) reported a case where COVID-19 appeared to accelerate CADASIL symptom onset. They proposed that the systemic inflammation and vascular stress caused by severe COVID-19 may have triggered an inflammatory cascade, leading to earlier or more severe clinical manifestations of CADASIL in their patient (Król et al., 2022).

Although CADASIL is classically described as a non-inflammatory small vessel arteriopathy, rare inflammatory-like presentations have been reported. Schiess et al. (2018) reported atypical CADASIL cases that clinically and radiologically mimicked MS or related demyelinating conditions. The cases presented unusual inflammatory features like intrathecal immune activity (elevated IgG index, unmatched OCBs), gadolinium-enhancing MRI lesions suggesting active CNS inflammation, and spinal cord involvement. The authors hypothesized that mutant *NOTCH3* could cause degeneration of small cerebral arteries, potentially leading to focal ischemic damage. They also stated that this may compromise the blood-brain barrier (BBB), exposing brain antigens (normally hidden from the immune system) to circulating immune cells. Such exposure could potentially trigger an autoimmune response against CNS components, resulting in intrathecal IgG production, OCB formation, and demyelinating-like lesions (Schiess et al., 2018). This proposed mechanism may explain our patient’s acute occipital lesion with gadolinium enhancement (Figure 2G), subsequent gliosis without recurrent enhancement (Figure 2H), and progressive intrathecal IgG synthesis. Alternatively, the enhancement may reflect acute/subacute ischemia with associated BBB breakdown, but the inflammatory findings suggest a possible superimposed parenchymal inflammation.

Recent mechanistic studies by Panahi et al. (2023) have provided support to the hypothesis that CADASIL pathology can trigger immune responses within cerebral vasculature. They reported that the accumulation of mutant NOTCH3 protein in VSMCs leads to endoplasmic reticulum stress, resulting in vessel damage. Their histological analyses revealed significant immune cell infiltration, activated microglia, and macrophages surrounding damaged cerebral arterioles. The VSMCs expressing mutant NOTCH3 showed increased interleukin-6 (IL-6) and intercellular adhesion molecule-1 (ICAM-1) expression. Additionally, extensive complement deposition (C3d and MAC/C5b-9) was identified on approximately 70% of affected arterioles, indicating alternative complement pathway activation (Panahi et al., 2023). Given these findings, the c.6102dup variant might similarly cause VSMC injury and subsequent immune activation in our patient. However, the inflammatory markers observed are not specific to CADASIL, and further studies are necessary to determine whether her inflammation is directly associated with the c.6102dup variant, as single-case observations alone cannot establish causation.

We also genetically confirmed that our patient’s 73-year-old mother carries the same *NOTCH3* c.6102dup (p.Gly2035Argfs*60) variant. Two decades earlier, the mother who also has migraines with aura, had been diagnosed with MS based on non-specific neurological symptoms and diffuse WMHI (Figures 3A–C). Despite receiving standard MS treatments, her condition progressively worsened, leading to persistent cognitive and motor decline without a typical relapsing-remitting course (McGinley et al., 2021). She experienced multiple bouts of further impairments in her 60s and is currently wheelchair-bound. Her MRI findings showed extensive confluent T2-weighted WMHI predominantly involving the corona radiata and subcortical white matter, characteristically the external/extreme capsule consistent with CADASIL (Figures 3A, C). However, similar to our index patient, she is also lacking anterior temporal lobe white matter involvement (Figure 3B). Although CSF results from her original MS diagnosis (20 years prior) are unavailable, her neurologist verbally confirmed the lack of abnormalities in MS-specific markers. Confirmatory GOM testing was not performed due to the mother’s advanced age and personal reluctance toward invasive procedures, limiting diagnostic certainty.

The inheritance pattern observed in this family suggests a possible autosomal dominant transmission (the known inheritance pattern for CADASIL), since the variant is present in both the patient and her mother across two confirmed generations. However, as the variant remains classified as a VUS, definitive conclusions about inheritance cannot yet be made. The patient does not have siblings. Additionally, the patient’s deceased maternal grandmother developed dementia of unclear etiology in her early 80s (a known manifestation of CADASIL), but no genetic testing was performed. While her dementia raises suspicion for familial CADASIL, the link to this variant remains uncertain. Our patient’s eldest child, a 20-year-old male, tested negative for CADASIL and had normal MRI findings. A younger, non-adult female child has not undergone genetic testing due to ethical considerations. However, given her strong history of migraines with aura and family history of the variant, she may have an increased likelihood of carrying the particular *NOTCH3* variant. Migraine with aura commonly precedes other CADASIL symptoms and typically presents earlier in females (peak age 16–30) compared to males (peak age 31–40) (Guey et al., 2015). Additional familial segregation analyses are needed to clarify the variant’s pathogenicity and clinical significance in this family. In addition, we do not have a skin biopsy on any of the family members.

As of November 2024, our patient is effectively managed with dual antiplatelet therapy (aspirin and cilostazol), atorvastatin for secondary stroke prevention, and verapamil for migraine prophylaxis (Figure 1). Her neurological exam remains stable, with complete resolution of visual symptoms. Our patient appreciates the extensive work-up that went into making a diagnosis, the care that she has received and the relatively good course that her CADASIL disease has taken so far. She particularly is thankful for the improvement in the frequency and severity of her migraine headaches. However, persistent inflammatory markers in both CSF and serum indicate potential unmet therapeutic needs for inflammation management, either during acute episodes or prophylactically. Should an autoimmune mechanism be established, targeted treatments could benefit patients with confirmed pathogenic *NOTCH3* variants presenting with inflammatory features.

## Data Availability

The original contributions presented in this study are included in this article/supplementary material, further inquiries can be directed to the corresponding author.
